# Perspectives on phase engineering of nanomaterials

**DOI:** 10.1093/nsr/nwae289

**Published:** 2024-08-20

**Authors:** Zhenyu Shi, Yuxuan Wu, Xinyang Ruan, Wei Zhai, Zijian Li, Li Zhai, An Zhang, Hua Zhang

**Affiliations:** Department of Chemistry, City University of Hong Kong, China; Department of Chemistry, City University of Hong Kong, China; Department of Chemistry, City University of Hong Kong, China; Department of Chemistry, City University of Hong Kong, China; Department of Chemistry, City University of Hong Kong, China; Department of Chemistry, City University of Hong Kong, China; Hong Kong Branch of National Precious Metals Material Engineering Research Center (NPMM), City University of Hong Kong, China; Department of Chemistry, City University of Hong Kong, China; Department of Chemistry, City University of Hong Kong, China; Hong Kong Branch of National Precious Metals Material Engineering Research Center (NPMM), City University of Hong Kong, China; Hong Kong Institute for Clean Energy, City University of Hong Kong, China; Shenzhen Research Institute, City University of Hong Kong, China

## Abstract

This perspective highlights the representative progress of phase engineering of nanomaterials (PEN) with an emphasis on noble metals and transition metal dichalcogenides, and proposes future directions in this emerging field.

The phase, describing the atomic arrangement of nanomaterials, has emerged as one of the key structural parameters in determining their properties and functions [1]. Recently, great progress has been made towards the phase engineering of nanomaterials (PEN) and a wide variety of nanomaterials with unconventional crystal phases, amorphous phases and heterophases with diverse physiochemical properties have been prepared [2]. The PEN strategy offers a new feasible and effective way to prepare novel functional nanomaterials towards various applications. In this perspective, we will summarize the representative work related to PEN with a particular emphasis on noble metals and transition-metal dichalcogenides (TMDs). Finally, we will provide our personal insights on the challenges and future research directions in this emerging field.

## PHASE ENGINEERING IN NOBLE-METAL NANOMATERIALS

**Figure 1.**
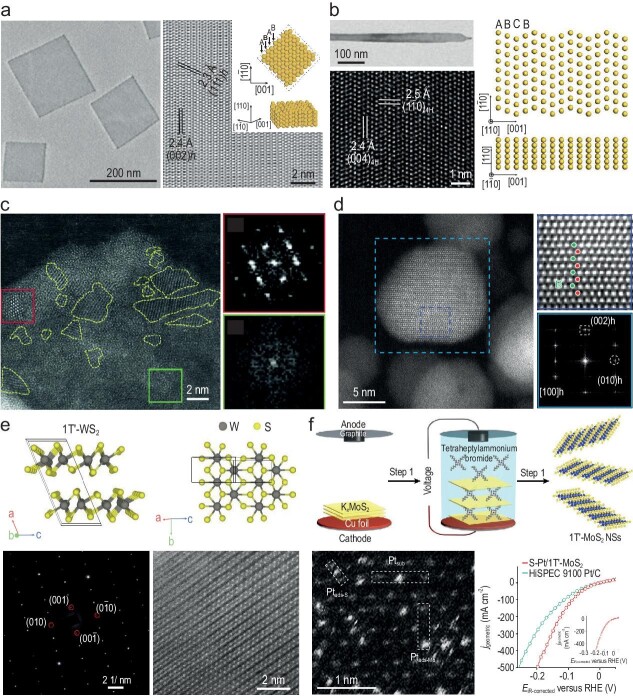
(a) Transmission electron microscopy (TEM) (left panel) and high-resolution TEM (HRTEM, right panel) images of 2H-Au nanosheet. The inset shows the atomic structure of the 2H-Au. Reproduced with permission from [3]. Copyright 2011 Springer Nature. (b) TEM image (left-top panel), HRTEM image (left-bottom panel) and schematic illustration (right panel) of 4H-Au nanoribbons. Reproduced with permission from [4]. Copyright 2015 Springer Nature. (c) TEM image and the corresponding fast Fourier transform (FFT) patterns showing the crystalline and amorphous parts of the Pd nanosheets. Reproduced with permission from [8]. Copyright 2018 Wiley-VCH. (d) High-angle annular dark-field scanning transmission electron microscopy (HAADF-STEM) images and the corresponding FFT pattern of 2H-Pd nanoparticles. Reproduced with permission from [9]. Copyright 2020 American Chemical Society. (e) Schematic illustration (top panel), selected area electron diffraction pattern (bottom-left panel) and HAADF-STEM image (bottom-right panel) of a typical 1T′-WS_2_ crystal. Reproduced with permission from [15]. Copyright 2021 Springer Nature. (f) Schematic illustration (top panel) showing the preparation process of 1T′-MoS_2_ nanosheets, atomic-resolution HAADF-STEM image of s-Pt/1T′-MoS_2_ (bottom-left panel) indicating that three kinds of Pt atomically dispersed on 1T′-MoS_2_ NS and polarization curves (bottom-right panel) revealing the catalytic performance of the synthesized s-Pt/1T′-MoS_2_ catalyst towards HER as compared with the commercial Pt/C. Reproduced with permission from [17]. Copyright 2023 Springer Nature.

Currently, noble-metal nanomaterials with unconventional phases can be prepared via either the direct-synthesis or the phase-transformation strategies [1]. The direct-synthesis strategy involves template-assisted synthesis, wet-chemical reduction, seed-mediated epitaxial growth methods and so on [2]. By using graphene oxides as templates, our group for the first time reported the synthesis of Au square sheets (AuSSs) with the unconventional 2H-type hexagonal close-packed (*hcp*) phase [3] (Fig. 1a). Subsequently, our group also prepared Au nanoribbons with the unconventional 4H phase by using a wet-chemical reduction method [4] (Fig. 1b). Moreover, by carefully tuning the experimental conditions, we have synthesized a series of Au nanomaterials with heterophases including 4H/face-centered cubic (*fcc*) Au nanorods [5], 2H/*fcc* Au nanosheets [6] and *fcc*-2H-*fcc* Au nanorods [7], and unconventional-phase Pd nanomaterials including amorphous/crystalline heterophase Pd nanosheets [8] (Fig. 1c) and amorphous Pd nanoparticles [9] via wet-chemical synthesis. Moreover, by using unconventional-phase noble metals as seeds, a library of noble-metal nanostructures such as Ag, Pd, Pt, Rh, Ru, Ir and Os with *fcc*-2H-*fcc*, 4H/*fcc* or 2H/*fcc* heterophase structures [9–12] can also be prepared through the seed-mediated epitaxial growth route.

The phase-transformation strategy is another effective way to prepare unconventional-phase noble-metal nanostructures that are normally difficult to prepare by using direct-synthesis methods. This strategy includes thermal activation [9], surface modification [11], electron/ion beam irradiation [13] and many other methods. For example, our group found that amorphous Pd nanoparticles could be transformed into 2H-Pd nanoparticles by using the thermal-activation method [10] (Fig. 1d). Besides, we observed that the 2H-Au nanosheets could be transformed into the *fcc* phase after exchanging the surface oleylamine molecule with 1-dodecanethiol [11]. In addition, electron-beam irradiation could precisely induce the phase transformation of noble metals in a specific small area [13]. For instance, it was reported that electron-beam-irradiation treatment could completely transform a 6.8-nm Au nanoparticle, which was attached to the 4H nanodomain of the Au nanorod, from the *fcc* phase to the 4H phase [13].

## PHASE ENGINEERING IN TMD NANOMATERIALS

The crystal phase is one of the key factors determining the physicochemical properties of TMD nanomaterials. Unlike the thermodynamically stable 2H-MoS_2_, which is a semiconductor, the unconventional 1T-MoS_2_ and 1T′-MoS_2_ exhibit metallic and semi-metallic properties, respectively, showing great potential for various applications including electronics, electrocatalysis, energy storage, etc. [2]. However, the preparation of TMDs with unconventional phases remains a great challenge. By using the traditional Li-intercalation, colloidal/hydrothermal synthesis, laser-treatment, heteroatom doping and other methods, mixed-phase TMDs (i.e. 2H phase mixed with 1T phase, 1T′ phase, etc.) can be easily obtained [1,2], while the intrinsic properties of the unconventional-phase TMDs remain vague. Chemical vapor deposition (CVD) has been used to prepare 1T′-MoS_2_ monolayers and heterophase bilayers [3] and our group recently reported the growth of 1T′-MoS_2_ nanoribbons on the top of 1H-MoS_2_ nanosheets, forming vertical 1H/1T′ MoS_2_ heterophase structures [14]. However, the low yield and harsh experimental conditions of the CVD method limit its practical application.

Recently, the emerging gas–solid synthesis has evolved as one of the most effective approaches to prepare high-quality and high-phase-purity TMDs with unconventional phases [2]. For example, our group successfully synthesized a series of high-purity 1T′-phase group VIB TMD crystals, including MoS_2_, MoSe_2_, WS_2_, WSe_2_, MoS_2_*_x_*Se_2(1−_*_x_*_)_ and WS_2x_Se_2(1−_*_x_*_)_, by employing the gas–solid reaction method [15]. The atomic structure of 1T′-WS_2_ is shown in Fig. 1e. Furthermore, our group reported a simple, effective and universal salt-assisted synthesis strategy to prepare gram-scale 1T′-TMD crystals using thermodynamically stable commercial TMD powders and easily accessible metal salt [16]. Then, the chemical/electrochemical intercalation method could be used to prepare high-phase-purity 1T′-TMD nanosheets in solution. Very recently, by using an optimized electrochemical intercalation method, our group successfully prepared 1T′-MoS_2_ nanosheets with high phase purity and micrometer size, and a thickness of 1.4 ± 0.4 nm [17] (Fig. 1f, top panel). Impressively, 1T′-MoS_2_ has been used as a template to grow single-atomically dispersed Pt (s-Pt) atoms with Pt loading of ≤10.0 wt% (Fig. 1f, bottom-left panel), exhibiting extraordinary activity and stability for hydrogen evolution reaction (HER) (Fig. 1f, bottom-right panel). Our work clearly demonstrates that 1T′-TMDs can be ideal supports for catalysts used for various important reactions including water electrolysis. In order to improve the stability of 1T′-TMDs, by using 4H-Au nanowires as templates, our group synthesized high-phase-purity and stable 1T′-TMD monolayers, including WS_2_, WSe_2_, MoS_2_ and MoSe_2_, via a facile and rapid wet-chemical method [18]. Impressively, the as-synthesized 4H-Au@1T′-TMD core–shell nanowires have been used for ultrasensitive surface-enhanced Raman scattering detection.

## CHALLENGES AND PERSPECTIVES

With the rapid development of PEN during recent decades, a wide range of nanomaterials beyond noble metals and TMDs have also been prepared with unconventional phases, such as metal oxides, carbides, etc. [2]. For example, IrO_2_ with unconventional 1T and 3R phases can be prepared through the mechano–thermal synthetic processes, and molybdenum carbide nanomaterials with unconventional cubic and hexagonal phases are obtained via a high-temperature heat-treatment strategy [2]. However, many challenges still exist in this emerging field. First of all, the formation mechanism of the unconventional phase in nanomaterials remains unclear. Currently, the preparation of these unconventional-phase nanomaterials heavily relies on the researchers’ experience and there is no general theory or principle that can guide the phase-engineering experiments [1,2]. Second, the controlled preparation of nanomaterials with unconventional phases is still challenging. The composition, morphology, size, dimension, facets and other parameters of the unconventional-phase nanomaterials cannot be precisely controlled during their preparation. Third, the metastable nature of unconventional-phase nanomaterials might make it difficult but more compelling for both fundamental research and application [17]. The phase or atomic arrangement of these metastable nanomaterials could be changed or even destroyed during the structure characterization or property study, which seriously hampers the investigation of their precise structures and intrinsic properties. Moreover, the nanomaterial-based devices that work in a practical application could undergo a continuous and irreversible phase transition, which may result in the degradation or deterioration of the nanomaterial structure and performance in the long term.

Based on the current research status and the aforementioned challenges, we propose some future research directions in PEN. First of all, chemical synthesis could be considered as the ‘(self-)assembly of atoms’ via chemical bonding by using atoms as ‘building blocks’, which is different from the concept of normal (self-)assembly that is widely used to prepare superstructure/superlattice materials using different building blocks such as nanoclusters, nanomaterials, micromaterials, etc. [19]. With the powerful PEN strategy, various nanomaterials with unconventional phases have been prepared. For example, after Au precursors were reduced to Au atoms, not only normal *fcc* Au nanomaterials with various morphologies, architectures, sizes, dimensions, facets, etc., but also unconventional hexagonal 2H-AuSSs [3], 4H-Au nanoribbons [4], 2H/*fcc* Au nanoplates [20] and nanosheets [6], 4H/*fcc* Au nanorods [5] and *fcc*-2H-*fcc* Au nanorods [7] have been obtained based on the ‘(self-)assembly’ of Au atoms under different reaction conditions. In the future, how to use the PEN strategy to prepare rationally designed ‘artificial atomic-assembled structures (AAASs)’ that could show unique novel physicochemical properties, functions and applications, especially for large-scale preparation via chemical synthesis, is extremely important. Second, it is highly desired to conduct in-depth and systematic studies to uncover the formation mechanism of unconventional phases by using cutting-edge technologies. For example, *in situ*/*operando* characterization techniques such as *in situ* transmission electron microscopy (TEM)/scanning transmission electron microscopy (STEM), *in situ* X-ray absorption spectroscopy, *operando* Fourier-transform infrared spectroscopy, *operando* Raman spectroscopy, etc. could be used to monitor the evolution of atomic arrangements and electronic structures during the formation of unconventional phases. With the combination of advanced characterization techniques, computational calculations and theoretical studies, the formation mechanism of unconventional phases is expected to be unraveled in the future, which could provide constructive guidance on the controlled synthesis of nanomaterials with unconventional phases. Third, various general and scalable preparation methods should be developed for preparing nanomaterials with unconventional phases. Thriving artificial intelligence techniques could bring new ideas to the design and controlled synthesis of such nanomaterials in a more efficient way. In the meantime, a comprehensive structure–property–performance relationship between the unconventional-phase nanomaterials needs to be established, which could in return guide researchers in preparing novel nanomaterials with desired properties and functions for specific applications. Fourth, it is of great importance to develop new strategies that could enhance the stability and durability of unconventional-phase nanomaterials in order to ultimately use these materials in practical applications.
